# Supplementary material for: Single Cell Transcriptomic and Chromatin Profiles Suggest Layer Vb Is the Only Layer With Shared Excitatory Cell Types in the Medial and Lateral Entorhinal Cortex
Source: Front Neural Circuits. 2022 Jan 26;15:806154. doi: 10.3389/fncir.2021.806154 (PMC8826650; doi:10.3389/fncir.2021.806154)
Supplement: Supplementary file 14 [file Data_Sheet_1.ZIP › LEC_Sup_vs_MEC_Sup_Motifs/homerResults/motif2.similar.html]

motif2

## Information for motif2

G
A
T
C
G
A
C
T
C
T
G
A
G
C
T
A
C
G
A
T
C
G
T
A
C
T
G
A
G
A
C
T
C
T
G
A
C
A
T
G
  
Reverse Opposite:  

G
T
A
C
A
G
C
T
C
T
G
A
G
A
C
T
G
C
A
T
C
G
T
A
C
G
A
T
G
A
C
T
C
T
G
A
C
T
A
G
  

|  |  |
| --- | --- |
| p-value: | 1e-120 |
| log p-value: | -2.777e+02 |
| Information Content per bp: | 1.667 |
| Number of Target Sequences with motif | 2301.0 |
| Percentage of Target Sequences with motif | 17.26% |
| Number of Background Sequences with motif | 2099.9 |
| Percentage of Background Sequences with motif | 10.54% |
| Average Position of motif in Targets | 264.8 +/- 201.8bp |
| Average Position of motif in Background | 340.2 +/- 277.9bp |
| Strand Bias (log2 ratio + to - strand density) | -0.0 |
| Multiplicity (# of sites on avg that occur together) | 1.11 |
| Motif File: | file (matrix) reverse opposite |

### Similar de novo motifs found

|  |  |  |  |  |  |  |  |
| --- | --- | --- | --- | --- | --- | --- | --- |
| Rank | Match Score | Redundant Motif | P-value | log P-value | % of Targets | % of Background | Motif file |
| 1 | 0.893 | C G A T C A G T G A T C A G C T C G T A C G A T C G T A C G T A C G T A C G A T C G T A C T A G | 1e-113 | -262.325475 | 17.69% | 11.05% | motif file (matrix) |
| 2 | 0.643 | G C A T C T G A A G T C G C A T C T A G G C T A A G C T G C T A C G T A G T C A | 1e-101 | -234.160664 | 57.87% | 48.59% | motif file (matrix) |
| 3 | 0.762 | C T G A G A C T G C A T C T G A C G T A A G C T G C T A T C A G | 1e-99 | -228.010816 | 41.46% | 32.69% | motif file (matrix) |
| 4 | 0.617 | G C A T G T C A G C T A C G A T G A C T C G T A C A T G G T A C C A T G T G A C | 1e-72 | -166.796566 | 55.50% | 47.69% | motif file (matrix) |
| 5 | 0.825 | A G C T C G T A C G T A G T C A C G T A C G T A A C G T C G T A | 1e-52 | -120.369317 | 31.62% | 25.70% | motif file (matrix) |
| 6 | 0.669 | C T G A T A G C C G T A G T C A A C G T A G C T C T G A C A T G C G A T C T G A | 1e-47 | -109.808509 | 10.73% | 7.25% | motif file (matrix) |
| 7 | 0.702 | G A T C G C A T T C G A C G A T A G C T A G C T C G T A T A G C C G T A C A T G | 1e-40 | -93.428545 | 15.83% | 11.91% | motif file (matrix) |
| 8 | 0.710 | T G A C A G T C G C T A C G T A C G T A C G T A C G T A A C G T C G T A C T A G | 1e-34 | -80.015487 | 8.91% | 6.17% | motif file (matrix) |
| 9 | 0.744 | A C G T C G T A C A T G A G T C G C A T C G T A C G A T C G A T C G A T G C A T G A C T C G T A | 1e-29 | -66.877982 | 8.45% | 6.00% | motif file (matrix) |
| 10 | 0.647 | C A G T A G T C A C G T C G T A A C G T A C G T C G T A A C T G | 1e-23 | -54.352108 | 19.30% | 16.00% | motif file (matrix) |
| 11 | 0.624 | A C G T A C G T C G T A C G T A A C G T C G T A C G T A C G T A | 1e-16 | -38.418176 | 9.10% | 7.16% | motif file (matrix) |
| 12 | 0.675 | C G T A C G T A A C G T G T C A C T G A C G T A G T C A A C G T | 1e-8 | -19.237686 | 54.17% | 51.68% | motif file (matrix) |
